# Hospital Meetings, &c.

**Published:** 1900-06-09

**Authors:** 


### HOSPITAL MEETINGS, &C.

### HOSPITAL FOR SICK CHILDREN, GREAT ORMOND STREET.

In the absence of the Duke of Fife, the president, the chair was taken at the forty-eighth annual Court of Governors of the Hospital for Sick Children, Great Ormond Street, by Mr. Arthur Lucas, the chairman of the Committee of Management, on May 30th.

After the proceedings of the Committee of Management had been duly confirmed, the report and accounts for 1899 were presented. The ordinary income for the year was £11,477. The extraordinary income was £3,097 (legacies, £2,597; donations towards theatre improvements, £500). In addition, there was received and invested £3,230 on account of the Endowment Fund. The extraordinary expenditure was £3,408, of which £2,657 was on the nurses' house, £331 on the operating theatre improvements, and £300 on other buildings. At the festival dinner, when Lord Peel presided, £5,440 was collected. The committee gratefully acknowledged a third donation of £500 from the Prince of Wales's Fund to enable them to open the whooping cough ward. The opening of this has been delayed by the necessity for using the adjoining small ward as a temporary operating room while the improvements were being carried out in the theatre. Emphasis was laid on the fact that the new ward would cost at least £1,000 a year to maintain, and that of this only half was provided from the Prince's Fund. A double benefaction had during the year been conferred on the hospital by Sir Theodore Martin as a memorial of the late Lady Martin (Helen Faucit). He had given £1,000 to found a cot, and £500 for the reconstruction of the operating theatre, which had been found quite inadequate for their present needs. They had now been enabled to raise the theatre to the best standard of modern requirements. The walls had been lined with marble; a terrazzo floor had been laid down; an efficient sterilising apparatus had been supplied; new glass and marble fittings had been provided. Indeed, the committee had endeavoured to carry out all the improvements suggested by the surgeons as necessary for the success of the operations to be performed. During the year the building next door, which formed part of the Convent of St. John and St. Elizabeth, purchased in 1898, had been converted into rooms for the nurses, and forty of them were now installed in it. This change had been most beneficial, and had produced thoroughly satisfactory results in the increased health and comfort of the staff. The adaptation of the premises was mainly executed by their works department at a cost of £4,672, and included the entire reconstruction of the drainage; the introduction of baths and lavatories and an adequate water supply; the heating and ventilation of the passages, the installation of the electric light, and the division of the dormitories into separate rooms and cubicles. One great advantage of the acquisition was the garden at the back—an invaluable boon both to the children and the nurses; £24,000 was to be borrowed on mortgage to meet the final instalment of £20,000 of the purchase money, and repay £4,000 to the hospital account—the cost of adapting and filling the nurses' home. The interest on this debt is paid out of the nurses' fees, and the committee appealed for help in paying off at least £1,000 a year. The new in-patients during the year numbered 1,962, the outpatients 23,892, and the patients sent to Cromwell Home, 213. The out-patients' attendances numbered 94,156. The cost of each in-patient per week was £1 10s. 11½d.; of each outpatient, 1s. 10¾d. Twenty-two nurses were now on the books of the private nursing staff, and this branch of the work of the hospital had made excellent progress during the year, the supply being frequently inadequate to the demand for services. Particular attention was drawn to the working guild founded by Mrs. John Murray. The president of the guild was the Duchess of Fife. The object was to supply the clothes needed by the children in the hospital. During the year over 960 garments were thus provided, making close on 6,000 in the seven years since the society was founded. In connection with the administration of the hospital, it was recorded that Viscount Peel had been elected a vice-president; and that Sir Charles Crawford and Sir Cameron Gull having resigned their seats on the committee of management, Mr. Roger Cunliffe and Mr. Edward L. Somers Cocks had been elected to fill the vacancies. After twenty-four years of invaluable services Dr. Thomas Barlow, the senior physician, had been obliged to resign his appointment owing to the pressure of other work. He had been elected a consulting physician, and the committee were proud that in this way his name would still remain on the list of the staff. Dr. A. E. Garrod had been elected a physician, and his place as assistant physician had been filled by the election of Dr. G. F. Still; Dr. Thursfield had been elected medical registrar in his place. Mr. Templeton having resigned the post of surgical registrar, Mr. Wallace had been elected in his place. Thanks were tendered to Mr. B. Dyball on his resignation as medical superintendent, and Mr. R. G. Ralston, F.R.C.S., had been elected in his place.

In moving the adoption of the report, the Chairman expressed regret that the Duke of Fife had been prevented by other engagements from presiding that day, because it was to the advantage of the institution that members of the Royal Family should be present on such occasions. The history of the hospital during the last 12 months had been an extraordinary one. They had been in the depths of despair; they were now full of hope, and in the happy position of seeing their way to carry on the work of the hospital for the next 12 months uninterruptedly. Very briefly stated, their position was that they spent generally about £16,000 a year, while the income they could fairly rely on from subscriptions, donations, and other sources amounted only to about £9,000. This left them, therefore, with an annual deficit of about £7,000. Generally they were able to meet this by appropriating legacies to current account. This, he admitted, was bad finance, but if they were sinners in this respect they sinned in company with the other London unendowed hospitals. Last year, however, legacies failed them—amounting only to £2,597—and at the end of the year they owed their bankers £4,500. This, with the prospective customary deficiency of £7,000, convinced the committee that by the end of the present year they would be in debt to the extent of £11,000 or £12,000. This was a bad state of things, and they had very seriously to consider what should be done. It might be said why did they not close part of the wards and receive fewer patients. But the closing of a ward really meant a very small comparative saving. The main expenses—servants, lighting, heating, and administration would still go on just the same. The only alternative of that kind was to close the whole hospital for a time, and that no doubt would arrest the attention of the public. But it was a dangerous step to take. Closing a hospital was not like closing a house. It took many years to collect and organise the skilled staff of nurses and medical men who carried on the work. Before entertaining such an idea they were bound to do all they could to avert so great a calamity. Their first notion was a special dinner, but they soon came to the conclusion that it was not possible to raise the necessary amount in this way because of the various funds engaging public charity. Then it occurred to a lady friend that if a good story could be told in a good paper the public would at once subscribe. She went to Mr. Anstey Guthrie and asked him to undertake the task, and this, with the consent of his editor, Mr. Burnand, he did. When the story appeared in *Punch* the proprietors, Messrs. Bradbury, Agnew, were so touched that they sent for Mr. Hope (the secretary), bombarded him with questions, and after having examined the accounts they intimated to them their intention to see the thing through and to put the hospital on a proper footing. As a result no less than £16,400 was subscribed. Of this £2,810 had to be invested, leaving £13,590 to be handed over to the treasurer. Their first step was to repay the £4,500 owing to their bankers, and they were now in a position to meet the expenses of the current year and still have £2,000 or so to the good for next year. This was a great and noble work done for the hospital, and the committee were filled with the deepest gratitude for it. They watched over the expenditure of the funds very jealously. There seemed to be an idea that they were well to do, but when he pointed out that their income from endowed funds was only about £1,000 a year, while their expenditure was £16,000, it would be seen that this was not so. There was also abroad a most erroneous idea that they were extravagantly managed. He could assure them, on his honour, that this was not so. He invited anyone to go into their household accounts and salaries and defied him to put his hand on any source of extravagance. People saw there was an air of luxury about the place, but that air of luxury and refinement was due to the individual taste and generosity of the sisters in the different wards who spent part of their fortunes on flowers and other matters which, while not necessaries, did much to add to the comfort of the patients. He could assure them that the committee considered themselves as only trustees of public funds, and they undertook to guard them most jealously.

The report was adopted, and Sir Wm. Agnew's name was added to the list of vice presidents.

The Chairman then moved the authorisation of the expenditure by the committee of £1,312 on new cisterns, mains, ward sinks, &c., reminding the meeting that by a very wise rule the committee could not spend more than £500 in construction without the sanction of a general meeting. The motion was adopted without discussion.

Mr. Justice Kekewich then moved a vote of thanks to Messrs. Bradbury, Agnew, and Co., proprietors of *Punch*, and to Mr. Burnand, the editor, for the appeal which had appeared in that paper. He was glad, he said, to pay a tribute to Mr. Guthrie—whom he knew chiefly as "Mr. Anstey"—who was a member of his own profession, and though he ought not to say he was glad he had not made it lucrative he was certainly glad he had turned to literature. The appeal in *Punch* had brought them subscriptions from all over the world—certainly from all over Europe, and the wide publicity would continue to be of great benefit to the hospital.

Mr. John Murray, in seconding the vote of thanks, observed that it was without precedent in their annals. The measure of their thankfulness was the depth of the despair they felt in December. Then everything looked so dark that their task seemed almost a hopeless one. It was impossible to add greater testimony to the power of sympathy than the way in which the work that *Punch* began had extended beyond the sphere of that paper. They had to thank most warmly Mr. Philip Yorke, the manager of the Palace Theatre, for the entertainment arranged and carried out so successfully. They had asked Sir William Agnew to become a vice-president, and Mr. Burnand and Mr. Yorke to be life governors, the only compliment in their power as a committee to pay; but they would always look back with gratitude on the immense service rendered just when it was so badly needed.

After the motion had been adopted, a resolution was carried placing the surgeon in charge of the diphtheria ward in the same category as the other medical officers of like standing in relation to the retirement rule; and votes of thanks to the medical officers, the auditors, and the chairman brought the proceedings to a close.

### MIDDLESEX HOSPITAL.

A quarterly Court of Governors of the Middlesex Hospital was held on the 31st ult., Sir Ralph Thompson presiding. Mr. F. Clare Melhado, the secretary-superintendent, read the report of the weekly board, which stated that they had adopted a scheme for utilising the space left vacant by the removal of the female cancer patients to the new wing formulated by a sub-committee. This would provide new kitchen offices and accommodation for additional nursing staff. In conjunction with the Medical Committee they had also approved the sub-committee's suggestion to provide further ward accommodation by erecting additional storeys over the projecting portion of the "Bird" Ward. Plans were also being considered for rearranging the laundry and for centralising the boiler and engineering departments. In connection with those schemes alterations were being made to the "Charles Street House" portion of the buildings to prepare it for the servants, who would be displaced from their present quarters in the old Cleveland Street houses; and sanction was asked for the expenditure of £98 on this. Approval was also invited of an estimate of £178 for a coal and food lift necessitated by the proposed conversion of the old cancer wards into kitchens. Negotiations were being carried on with the Berner's Estate Trustees for a lease of No. 5, Nassau Street, adjoining the new cancer wing. The report went on to state that Sir Ralph Thompson and Sir Arthur Watson had been re-elected chairman and deputy-chairman of the board respectively. Dr. Wm. Pasteur had been appointed for five years to take charge of the medical beds in Princess May Ward, and Dr. H. Campbell Thomson, the pathologist and curator, had been elected an assistant physician to fill the vacancy created by the appointment of Dr. Pasteur as full physician. Regret was expressed at the resignation of Mr. Storer Bennett from the office of dental surgeon, and it was recommended, in recognition of his long and valued services, he be appointed consulting dental surgeon to the hospital. Regret was also expressed at the death of Mr. George Howard, late hon. auditor, and for many years a governor; and of Miss Hall, late sister of "Founder" Ward, of enteric fever at Bloemfontein. Miss Hall retired from the nursing staff to join the Army Nursing Reserve, and was succeeded by Miss Froude, late night superintendent nurse in the cancer wing. Nurse Hobley, who had been twenty-one years in the service of the Hospital and Trained Nurses' Institute had retired, and was recommended for a pension of £18 3s. 4d. per annum. While gratefully acknowledging many generous donations during the past quarter, the board regretted to note that the total amount was nearly £500 less to the general funds than in the previous quarter of 1899. Special thanks were accorded to Mr. Lowry for writing, and to the editor of the *Morning Post* for publishing, an article entitled "The Valley of the Shadow," describing the work in the new cancer wards. This article had since been circulated as an appeal, and had produced a satisfactory response. The Metropolitan Drinking Fountain Association had kindly undertaken to erect a drinking fountain in the hospital garden. At the Convalescent Home the provisions for the "open-air" treatment of patients suffering from tuberculosis were working satisfactorily. During the quarter 163 patients and six nurses had enjoyed the benefits of the home. Sanction was asked for an expenditure of £290 for a new electric storage battery for the home. In the medical school Dr. Pasteur, having been appointed lecturer on medicine, had resigned his lectureship on practical medicine, and this had been filled by the appointment of Dr. Walter Essex Wynter. Two assistant physicians were to be associated with him as assistant lecturers. Arrangements had been made, and new rules for the lecturer on chemistry drawn up. During the quarter 1,103 medical and surgical patients were treated. Of these 73 died and 257 remained at the beginning of the new quarter. In the female cancer wing 29 new patients were admitted, and there were 13 deaths. In the out-patient department 11,543 were treated, the total attendances being 27,827, giving an average of 2·4 attendances for each patient, and an average of 305·8 patients seen daily.

The Chairman moved the adoption of the report, and referred to some of the items. They were asked, he said, to sanction part only of the scheme in contemplation; the remainder depended on a further report, and would be submitted to subsequent meetings. The key to the position was the need for increased laundry accommodation. In order to get that they must appropriate the present kitchens and put these somewhere else. It was admitted that the best place for kitchens was at the top of a building, and they proposed to take advantage of having moved the women's cancer ward to do this. They also intended to build an extra ward, so that the poor should not be damnified by the alteration. They were also increasing their nurses' accommodation. Their doctors continued to press for more nurses. The three houses in Cleveland Street must be pulled down by the terms of the leases, and they proposed to provide the extra accommodation there.

The report was adopted. The amounts in the almsboxes proved to be £98 19s. 9d., as compared with £65 12s. 9d. in the corresponding quarter last year, an increase on the quarter of £33 7s. This the Secretary-Superintendent stated was a record collection.

A vote of thanks to the chairman closed the proceedings.

### BROMPTON CONSUMPTION HOSPITAL.

The Earl of Derby presided at the fifty-ninth annual Court of Governors of the Hospital for Consumption, Brompton, on Thursday, 31st May ult. The secretary, Mr. W. H. Theobald, read the report of the Committee of Management for the past year. The total receipts were given as £35,683, of which legacies amounted to £14,099. Of 1,723 in-patients in the wards, and admitted during 1899, 1,175 were discharged, many greatly benefited; 258 had died, and there remained in the hospital 290. The average stay of each patient was 72 days. The daily average of beds occupied was 272. The new out-patients numbered 14,281, with 74,470 attendances. Dr. C. Y. Biss had resigned the position of physician, and had been succeeded by Dr. Hector Mackenzie. Dr. Sidney Martin had resigned the office of assistant physician, to which Dr. J. J. Perkins and Dr. Arthur Latham had been appointed. Dr. F. W. Price had been elected assistant resident medical officer, in the place of Dr. P. S. Hitchens resigned. Twenty-eight beds had been set apart for the "open-air" treatment in the south wing, and it was confidently hoped this would lead to satisfactory results. The new home for private and staff nurses was now ready for occupation. The site for the country branch and convalescent home had been engaging the unremitting attention of the committee, and subscriptions towards the building fund were greatly needed.

In moving the adoption of the report the Chairman remarked that they were entering upon their sixtieth year, and he did not think that if a popular vote were taken as to the continuance or withdrawal of the institution there would be a single vote for its withdrawal; on the contrary, there were very many who would bless the day they were brought in contact with its benefits. It was no disparagement to the hospital that, owing to the London climate, they were impelled to seek better conditions with regard to light and fresh air—the two great things which medical opinion was agreed upon that tubercular disease could be coped with. Brompton, as he and many others could well recollect, used to be a pleasant place among the fields. Now they were completely enclosed in bricks and mortar. But up till now the public subscriptions had been very slight indeed for the country branch. He put this down to the doubt they were in as to the site to be chosen and to the various funds now claiming attention. Many who would, no doubt, help them handsomely when a site had actually been chosen felt that they might hold their hand until it was settled. He hoped their friends would rally to their help. With regard to the new nurses' home, he felt sure that the better their nurses were housed and the nearer to the hospital the better would they be able to get the class of ladies they wanted for the work.

The report was adopted, and a vote of thanks terminated the meeting.

